# COVID-19 pandemic reclassification and implications for continuing uptake of COVID-19 vaccination: The case of Savannah Region, Ghana, 2023

**DOI:** 10.1016/j.ijregi.2025.100694

**Published:** 2025-06-27

**Authors:** Michael Rockson Adjei, Kwabena Adjei Sarfo, Cyril Kwami Azornu, Peter Gyamfi Kwarteng, Felix Osei-Sarpong, Janet Vanessa Baafi, Nana Akua Afriyie Bafana, Chrysantus Kubio, Sally-Ann Ohene, Martin Peter Grobusch

**Affiliations:** 1 Centre of Tropical and Travel Medicine, Department of Infectious Diseases, Amsterdam University Medical Center, location AMC, University of Amsterdam, Amsterdam, The Netherlands; 2 World Health Organization, Country Office, Accra, Ghana; 3 Ghana Health Service, Regional Health Directorate, Damongo, Ghana; 4 Ghana Health Service, East Gonja Municipal Hospital, Salaga, Ghana; 5 United Nations Children’s Fund, Country Office, Accra, Ghana; 6 Ghana Health Service, District Health Directorate, Odumase, Ghana; 7 Ghana Health Service, District Health Directorate, Kumasi-Oforikrom, Ghana; 8 Institute for Tropical Medicine, German Center for Infection Research (DZIF), University of Tübingen, Tübingen, Germany; 9 Institute of Infectious Disease and Molecular Medicine, University of Cape Town, Cape Town, South Africa; 10 Centre de Recherches Médicales en Lambaréné (CERMEL), Lambaréné, Gabon; 11 Masanga Medical Research Unit, Masanga, Sierra Leone

**Keywords:** COVID-19, Public health emergency of international concern, Post-pandemic, Savannah Region, Ghana, Vaccine acceptance

## Abstract

**Objectives:**

This study assessed the willingness of Ghanaians to accept COVID-19 vaccination following the pandemic reclassification. The findings are envisaged to facilitate implementation of contextual strategies to sustain the COVID-19 vaccination drive and avert case surges.

**Methods:**

A cross-sectional study was conducted in three districts (North Gonja, Sawla-Tuna-Kalba, and West Gonja) of the Savannah Region using an electronic questionnaire to interview respondents on willingness to accept COVID-19 vaccinations following the pandemic reclassification. Bivariate and multivariate logistic regression analysis were performed to identify factors associated with continuing COVID-19 vaccination uptake.

**Results:**

Respondents who would advocate for COVID-19 vaccination were more likely to accept it (adjusted odds ratio [AOR] = 8.4; 95% confidence interval [CI]: 6.18-11.41). Residing in an urban community (AOR = 0.78; 95% CI: 0.65-0.83) or having secondary education (AOR = 0.64; 95% CI: 0.46-0.89) was associated with reduced odds of COVID-19 vaccination acceptance.

**Conclusions:**

The willingness to accept COVID-19 vaccination was high among the respondents, and the majority would encourage their neighbors to receive vaccination. However, targeted approaches are required to address vaccine hesitancy among specific subpopulations, including urban communities and those with secondary-level education.

## Highlights

• COVID-19 pandemic reclassification has significant implications for vaccine uptake.
• Low vaccine acceptance was observed among the educated and urban inhabitants.
• Targeted communication strategies are required to improve uptake.
• Volunteerism is declining; acceptance of advocacy roles seems to be driven by incentives.

## Introduction

The COVID-19 pandemic followed a tortuous trajectory and defied predictions regarding its evolution in the initial phase [1]. The disease originated from Wuhan, China and spread to other parts of Asia, Europe, and the United States, and was declared a pandemic by the World Health Organization (WHO) in March 2020 [1]. As of December 31, 2023, approximately 773 million cases with seven million deaths had been recorded globally. Africa was one of the least impacted regions, accounting for approximately 1.2% (9.6 million) and 2.5% (175,000) of the global cases and deaths, respectively [2]. Ghana recorded its index cases on March 12, 2020, and as of December 31, 2023, nearly 172,000 cases and 1500 deaths had been documented [3].

Associated with the morbidity and mortality statistics were the economic and social disruptions that accentuated the impact of the pandemic. Millions of enterprises faced existential threats with downsizing of the labor force in some instances. Informal economy workers were particularly vulnerable because many lacked social protection and access to quality health care. These effects were heightened during the lockdown because of border closures, trade restrictions, and confinement measures, which interrupted domestic and international food supplies and reduced access to healthy, safe, and diverse diets [4].

To mitigate the impact of the pandemic, effective and safe COVID-19 vaccines were developed with remarkable speed and deployed with immediate goals of minimizing deaths, severe disease, and overall disease burden; curtailing the health system impact; fully resuming socioeconomic activity; and reducing the risk of new variant emergence [5]. As of November 2023, nearly 13.6 billion doses of COVID-19 vaccines had been administered globally, with 67% of the population completing primary series vaccination. About 646 million of the vaccine doses were used in Africa, and 33% of the population had completed primary series vaccination [2]. COVID-19 vaccines deployed in Ghana included AstraZeneca, Sputnik V, Moderna, Pfizer–BioNTech, and the Janssen COVID-19 vaccine. Nearly 28 million doses of the COVID-19 vaccine had been administered in the country as of November 2023, and 36.2% of the total population had completed the primary series vaccination (one dose for the Janssen COVID-19 vaccine and two for the other vaccine types) [6].

The objectives of the COVID-19 vaccine deployment were largely achieved. As of December 2022, COVID-19–related deaths and hospitalization had declined significantly; population immunity had improved; and the dominant circulating variant (Omicron sublineages) did not appear to be associated with increased severity [7]. While announcing that COVID-19 no longer constituted a public health emergency of international concern (PHEIC), WHO cautioned that the virus is “still killing, and it’s still changing” [8]. Continuing COVID-19 vaccine delivery is critical for pandemic prevention, preparedness, readiness, and response; and requires sustainable approaches that prioritize highest-risk individuals. WHO therefore urged countries to transition from emergency mode to integrating COVID-19 vaccination into routine systems [7].

Ghana launched the integration of COVID-19 vaccination into routine care in October 2023 with a primary goal of sustaining vaccine delivery among high-risk populations (including older adults, persons with underlying medical conditions, health care workers, and pregnant women) [9]. The uptake of COVID-19 vaccines is determined by demand- and supply-related factors. Risk perception, vaccine safety, and sociodemographic characteristics are among factors that influence uptake. Persons who believe that the vaccines are safe and those with higher perceived disease risk have increased likelihood of vaccination [10]. Exposure to accurate information, recommendation from trusted health care providers, and ease of access to vaccination services have also been found to be key determinants of uptake [10].

The declaration of the COVID-19 pandemic as no longer constituting a PHEIC was variedly interpreted by society, with implications for the continued uptake of COVID-19 vaccination.The objective of this study was to assess the willingness of inhabitants in three districts of the Savannah Region to accept COVID-19 vaccinations following the pandemic reclassification. The findings are envisaged to facilitate implementation of contextual strategies to sustain the COVID-19 vaccination drive and avert case surges.

## Methods

### Study design

**Figure 1:**
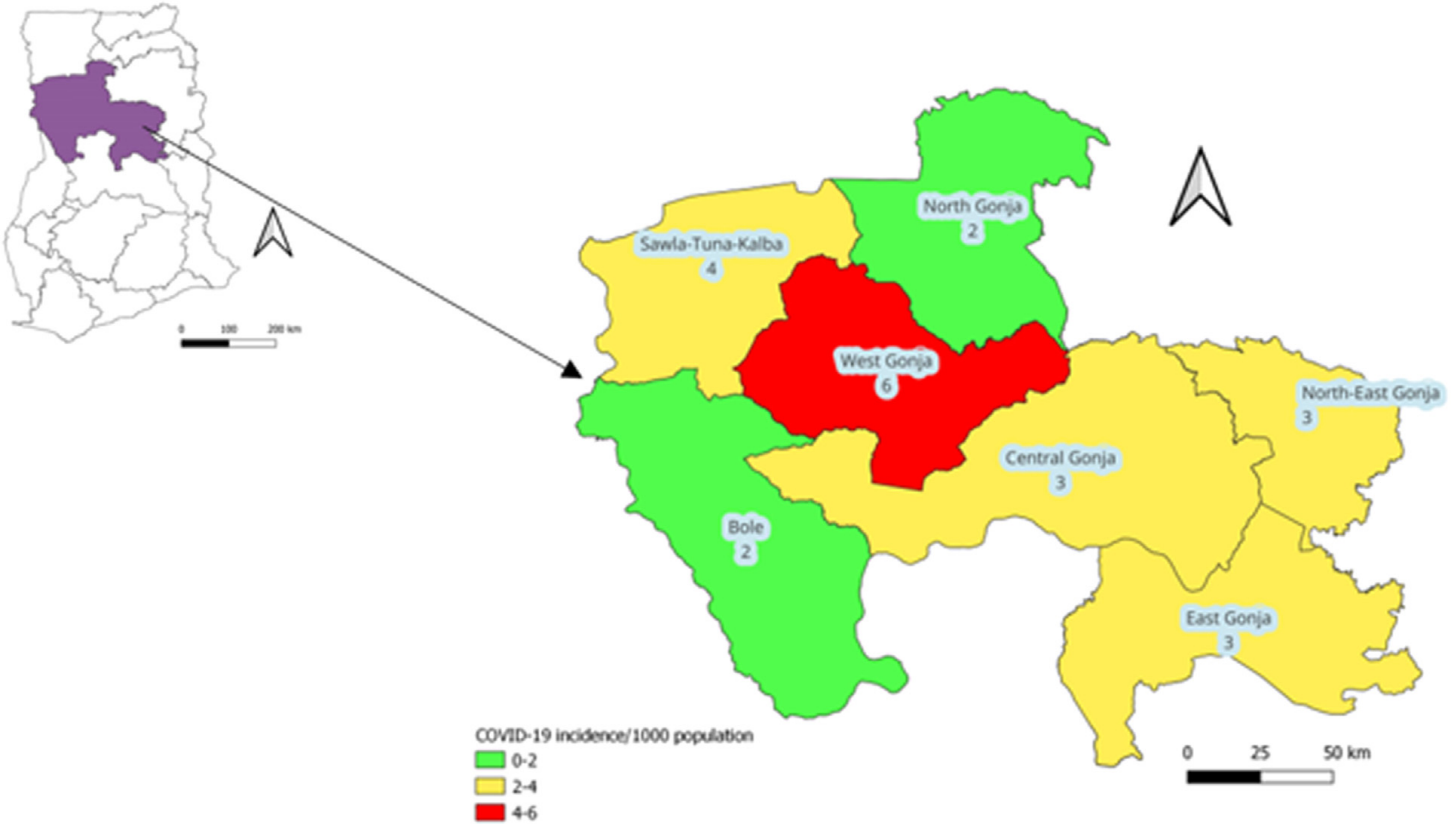
Incidence of suspected COVID-19 cases (per 1000 population), Savannah Region, January 2020-November 2023.

A cross-sectional study was conducted in three districts (North Gonja, Sawla-Tuna-Kalba, and West Gonja) of the Savannah Region from May to December 2023. The selection of the study districts was based on the incidence of suspected COVID-19 cases, classified as high (red), medium (yellow), and low (green) in Figure 1. North Gonja was selected over Bole to ensure sociocultural representativeness given that the latter shares similar characteristics with Sawla-Tuna-Kalba.

### Study setting

The Savannah Region was created from the Northern Region in 2019. It is bordered in the north by the Upper West and North East regions, in the west by Côte d’Ivoire and Burkina Faso, in the south by the Bono and Bono East regions, and in the east by Northern and Oti regions (Figure 1). It is the region with the largest land size in Ghana and occupies an area of 35,862 km^2^ with a total population of 649,627 according to the 2021 population and housing census [11]. Most of the inhabitants are peasant farmers pursuing grain farming and livestock rearing; most of the livestock farmers are undocumented nomads from neighboring countries [11].

There are seven administrative districts (Figure 1) and a total of 180 health care facilities comprising six hospitals, three polyclinics, 23 health centers, and 148 community-based health planning and services (CHPS) compounds. The region’s index COVID-19 case was confirmed in May 2020. As of November 2023, a total of 2574 cases (incidence: 3.8/1000 population) had been suspected, while 408 were confirmed (incidence: 0.6/1000 population), with three deaths (case fatality ratio: 0.7%) (Savannah Regional Health Directorate, unpublished). COVID-19 vaccination began in the region in March 2021, and as of November 2023, 332,135 vaccine doses had been administered. Approximately 79% and 46% of the target (persons aged ≥15 years) and general populations, respectively, had completed the primary series vaccination (Savannah Regional Health Directorate, unpublished).

The regional capital, Damongo, is in the West Gonja District. The district is predominantly urbanized with a population of 66,142 and 26 health facilities (West Gonja District Health Directorate, unpublished). As of November 2023, 418 (incidence: 6/1000 population) COVID-19 cases had been investigated, with 112 confirmed (incidence: 1.8/1000 population) (Savannah Regional Health Directorate, unpublished). Cumulatively, 40,433 doses of COVID-19 vaccines had been administered in the district. Approximately 87% of the target population (persons aged ≥15 years) and 50.5% of the general population had completed the primary series vaccination (Savannah Regional Health Directorate, unpublished).

Sawla-Tuna-Kalba borders Côte d’Ivoire and the Upper West Region and serves as a transit point for many travelers in the West African subregion. It is a transitional (mixed urban-rural) district with a population of 117,445 and 39 health facilities (Sawla-Tuna-Kalba District Health Directorate, unpublished). A total of 436 COVID-19 cases had been investigated (incidence: 4/1000 population) from the inception of the pandemic to November 2023, and 46 were confirmed (incidence: 0.4/1000 population) (Savannah Regional Health Directorate, unpublished). Nearly 89% of the target population (persons aged ≥15 years) and 51% of the general population had completed the primary series vaccination (Savannah Regional Health Directorate, unpublished).

North Gonja is predominantly rural and shares boundaries with Northern and North East regions. It has a total population of 64,039 with 18 health facilities (North Gonja District Health Directorate, unpublished). A total of 113 suspected cases (incidence: 2/1000 population), with 10 confirmed (incidence: 0.2/1000 population), had been recorded as of November 2023 (Savannah Regional Health Directorate, unpublished). The district had the highest uptake of COVID-19 vaccination in the region, with 118% and 68% of the target and general populations completing their primary series vaccination, respectively.

### Data collection

A multistage cluster sampling technique was used in selecting respondents in the study districts, ensuring representation of each subdistrict. A respondent was defined as any person aged 15 years and above who resided in the study area during the three (3) months preceding the study.

The communities (clusters) were selected using probability proportional to size, and 3-6 respondents were interviewed per community. In each cluster, the first household was selected by choosing a random direction from the community center by spinning a pen. Subsequent households were selected to the right of the first, with two houses skipped between each selection.

In the households, respondents were randomly selected, and individual written informed consent was obtained before interview. Privacy was ensured and the interview was conducted using an electronic questionnaire designed on KoboCollect (KoboToolbox, https://www.kobotoolbox.org/), an open-source data collection application.

Responses collected from the study participants included sociodemographic characteristics (including age, sex, place of residence, resident type, marital status, average monthly income, employment status, and educational level); COVID-19 vaccination status by card (complete primary series vs incomplete [received no dose or only one of a two-dose series]); whether the respondent would accept COVID-19 vaccination after the end of the public health emergency; and whether they would advocate for or encourage others to accept vaccination (advocacy position). Data were synced to a central server at the end of each interview.

### Data analysis

Data were extracted into a Microsoft Excel 16.0 spreadsheet and exported into Epi Info statistical software (Epi Info version 7.2.2.16; www.cdc.gov≥epiinfo) for analysis. Descriptive statistics (mean, median, standard deviation, range, first and third quartiles) were estimated for age. Frequency and percentage distributions of characteristics were computed using cross-tabulations to compare acceptance of COVID-19 vaccination post-emergency, dichotomized as “yes” and “no.” Independent variables that showed significant association with the outcomes (*P* <0.05) were included in the logistic regression model to identify factors associated with uptake of COVID-19 vaccination post-emergency. The results were presented as odds ratio at 95% confidence level.

## Results

Approximately 1% (2500/247,626) of the combined population of the study areas participated in the study, with 26.1% (653/2500) from North Gonja, 27.0% (675/2500) from West Gonja, and 46.9% (1172/2500) from Sawla-Tuna-Kalba. The mean age of the respondents was 34.6 years, with a standard deviation of 18.4 years. The youngest respondent was aged 15 years, while the oldest was 98 years. Twenty-five percent (625/2500) (first quartile [QI]) of the respondents were aged up to 24 years. Half (1250) of the respondents (median) were aged ≤32 years, while 75% (1875/2500) (third quartile [Q3]) were up to 42 years old. Most (57.8%) of the respondents were aged 20-39 years.

Approximately 57% (1434/2500) were female, and 79% (1974/2500) resided in urban communities. Only 17.1% (427/2500) were temporary (itinerant or nomadic) residents, and most (71.9%; 1798/2500) were married or cohabiting with their partners. Fifty-five percent (1376/2500) had no formal education (Table 1). Most (75.4%; 1884/2500) of the respondents were self-employed and engaged in farming or trading, and majority (85.9%; 2148/2500) earned an average monthly income of <100 USD. Approximately 72% (1788/2500) of the respondents had completed COVID-19 primary series vaccination (Table 1).

**Table 1:** Respondent characteristics, COVID-19 vaccine acceptance study, Savannah Region, Ghana, 2023.

Characteristic	COVID-19 vaccine acceptance post-emergency		Total N (%)	*P*-value
	Yes, n (%)	No, n (%)		
***Vaccination status (primary series)***				
Complete	1509 (84.4)	279 (15.6)	1788 (71.5)	0.222
Incomplete	586 (82.3)	126 (17.7)	712 (28.5)	
***District of residence***				
North Gonja	564 (86.4)	89 (13.6)	653 (26.1)	0.090
West Gonja	565 (83.7)	110 (16.3)	675 (27.0)	
Sawla-Tuna-Kalba	966 (82.4)	206 (17.6)	1172 (46.9)	
**Age (years)**				
15-19	251 (85.4)	43 (14.6)	294 (11.8)	**0.050**
20-29	634 (81.0)	149 (19.0)	783 (31.3)	
30-39	551 (83.1)	112 (16.9)	663 (26.5)	
40-49	329 (15.7)	47 (11.6)	376 (15.0)	
50-59	207 (84.8)	37 (15.2)	244 (9.8)	
≥60	123 (87.9)	17 (12.1)	140 (5.6)	
***Sex***				
Male	891 (83.6)	175 (16.4)	1066 (42.6)	0.843
Female	1204 (84.0)	230 (16.0)	1434 (57.4)	
***Place of residence***				
Rural	436 (82.9)	90 (17.1)	526 (21)	**0.002**
Urban	1508 (76.4)	466 (23.6)	1974 (79)	
***Resident type***				
Itinerant (Nomad)	367 (86.0)	60 (14.0)	427 (17.1)	0.211
Established (Permanent)	1728 (83.4)	345 (16.6)	2073 (82.9)	
***Marital status***				
Married/Cohabiting	1518 (84.4)	280 (15.6)	1798 (71.9)	0.193
Single/Widowed/Divorced	577 (82.2)	125 (17.8)	702 (28.1)	
***Education level***				
No formal education	1154 (83.8)	222 (16.1)	1376 (55.0)	**0.008**
Primary	394 (80.1)	98 (19.9)	492 (19.7)	
Secondary	424 (88.0)	58 (12.0)	482 (19.3)	
Tertiary	123 (82.0)	27 (18.0)	150 (6.0)	
***Employment status***				
Unemployed	392 (81.3)	90 (18.7)	482 (19.3)	0.254
Formal sector employee	114 (85.1)	20 (14.9)	134 (5.4)	
Self-employed	1589 (84.3)	295 (15.7)	1884 (75.4)	
***Income***				
<$100	1786 (83.2)	362 (16.8)	2148 (85.9)	0.062
$100-200	246 (86.9)	37 (13.1)	283 (11.3)	
>$200	63 (91.3)	6 (8.7)	69 (2.8)	
***Advocate for COVID-19 vaccination (advocacy position)***				
No	88 (44.7)	109 (55.3)	197 (7.9)	**0.000**
Yes	2007 (87.2)	296 (12.8)	2303 (92.1)	

**Figure 2:**
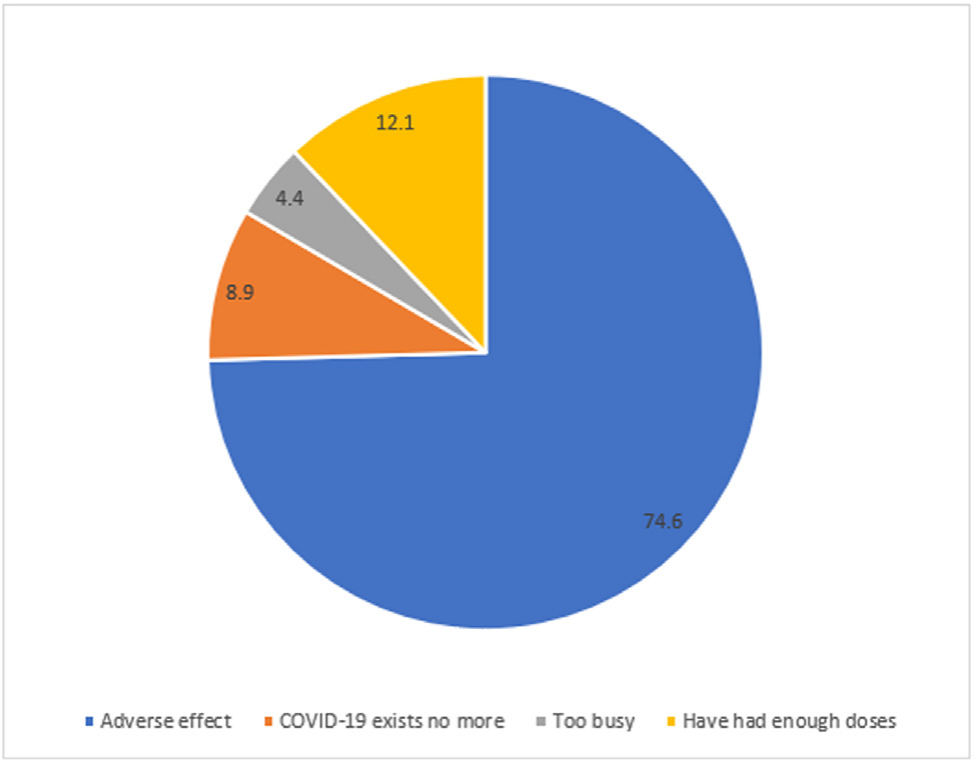
Reasons for COVID-19 vaccine refusal post-emergency, Savannah Region, Ghana, 2023.

Most respondents (83.8%; 2095/2500) stated that they would accept COVID-19 vaccination if made available, and 92.1% (2303/2500) indicated that they would advocate for or advise their neighbors to accept vaccination (Table 1). Nearly 75% (302/405) of those who would not accept vaccination cited anticipated adverse effects as the main reason (Figure 2).

In the regression model, place of residence, education level, and advocacy position were significantly associated with continuing acceptance of COVID-19 vaccination. Respondents who would advocate for or advise others to take the vaccine were more likely to accept vaccination (adjusted odds ratio [AOR] = 8.4; 95% confidence interval [CI]: 6.18-11.41). Residing in an urban community (AOR = 0.78; 95% CI: 0.65-0.83) or having secondary education (AOR = 0.64; 95% CI: 0.46-0.89) was significantly associated with reduced odds of vaccine acceptance (Table 2).

**Table 2:** Estimated odds ratio of COVID-19 vaccine acceptance, Savannah Region, Ghana, 2023.

Characteristic	OR	95% CI	*P*-value	Adjusted OR	95% CI	***P*-value**
***Place of residence***						
Rural	1.00			1.00		
Urban	0.92	0.88-0.96	0.002	0.78	0.65-0.83	**0.001**
***Education level***						
No formal education	1.00			1.00		
Primary	1.29	0.99-1.68	0.056	1.13	0.85-1.49	0.406
Secondary	0.71	0.52-0.97	0.031	0.64	0.46-0.89	**0.008**
Tertiary	1.14	0.73-1.77	0.557	0.87	0.53-1.43	0.579
***Advocate for COVID-19 vaccination (advocacy position)***						
No	1.00			1.00		
Yes	8.40	6.18-11.41	0.000	8.45	6.17-11.6	**0.000**

CI, confidence interval; OR, odds ratio.

## Discussion

We assessed predictors of COVID-19 vaccination uptake following the declaration of the COVID-19 pandemic as no longer constituting a PHEIC. The study was based on the health belief model (HBM), which postulates that health behavior of individuals is influenced by perceived threat of illness or disease (perceived susceptibility), belief of consequence (perceived severity), potential positive benefits of action (perceived benefits), perceived barriers to action, and exposure to factors that prompt action (cues for action) [12].

Risk perception influences health decision-making and tends to be elevated among individuals with lived experience or knowledge of someone who has been affected [13]. In the early phase of the pandemic, the perception of COVID-19 as a “rich man’s disease” was widespread, and many people living in Ghana underestimated the value of infection prevention measures [14]. However, following the surge in global cases and the confirmation of the country’s index cases, public health measures including restriction of movement were enhanced, and people began adopting positive health behaviors [15].

In this study, approximately 72% of the respondents had completed COVID-19 primary series vaccination, as opposed to the regional administrative coverage of 79%. It is noteworthy that these coverage rates are comparable (*P* = 0.324), supporting the representativeness of the study sample.

The COVID-19 vaccination coverage for the complete primary series in the Savannah Region exceeded the national average of 55.9% for persons aged ≥15 years [6]. This observation is contrary to the findings of Amponsah Tabi et al*.* [16], who compared the COVID-19 vaccination acceptance across the three zones of Ghana (northern, middle, and southern) and documented the uptake in the northern part as the lowest. The difference in the observations might be due to the timing, given that the study by Amponsah Tabi et al*.* [16] was conducted in 2021. Their findings might have prompted stakeholders to intensify efforts to generate demand for COVID-19 vaccination. The high coverage observed in our study might be an affirmation of the effectiveness of the remedial measures [6].

Many of the respondents indicated willingness to recommend COVID-19 vaccination to their neighbors, but a few were reluctant to receive the vaccine themselves. Given the concerns about the possible decline in uptake following the pandemic’s reclassification [17], this finding provides a promising prospect for sustaining the vaccination drive. Despite the goodwill, the reasons for refusing COVID-19 vaccination require urgent attention, considering that vaccine hesitancy is “infectious,” and a few hesitant persons could undo the effort of the majority [18]. The main reason cited—anticipated adverse effects—has also been documented in previous studies [[19], [20], [21]]. Other reasons, including “COVID-19 exists no more” and “I have had enough doses,” might stem from low risk perception, concerns about vaccine effectiveness, and the availability of other personal protective measures such as face masks and hand hygiene [22]. Conversely, persons who would encourage others to receive the vaccine but are reluctant themselves might be motivated by incentives such as allowances or social recognition, among others [23].

Community-based approaches have been demonstrated to improve COVID-19 vaccination uptake [24], and the willingness of more than 90% of the respondents to advocate for or advise a community member to get vaccinated is reassuring. According to the study by Udor et al*.* [25], individuals are more willing to accept COVID-19 vaccination when trusted community members advocate for or advise it. The observation that approximately 13% of respondents who would advocate for COVID-19 vaccination would not accept it themselves raises concerns about commitment, considering that advocacy is most effective when proponents lead by example [26,27]. It is possible that these respondents perceived the advocacy role merely as a form of employment rather than an altruistic endeavor [28]. Alternatively, these respondents may belong to the group that believes they have received a sufficient number of COVID-19 vaccine doses.

The finding that respondents living in urban communities were less likely to accept COVID-19 vaccination was contrary to the observation made by Udor et al*.* [25], who found no significant difference in the COVID-19 vaccine acceptance rate between rural and urban communities. The Udor et al. [25] study was conducted in 2021, a period when COVID-19 risk perception was substantial, and vaccination was in demand for personal protection and as a prerequisite for travel, employment, and access to some public places. Therefore, persons living in urban and rural communities were equally motivated to receive COVID-19 vaccination. However, these drivers of vaccination acceptance might have dissipated following decreased risk perception and the easing of the restrictions.

The low demand for COVID-19 vaccination in urban communities, as observed in our study, might be attributed to the rapid spread of misinformation and disinformation due to high internet penetration, particularly through social media where regulation and ethical constraint appear to be limited [29,30]. Although persons living in urban communities are more likely to be educated and have access to information, deciphering health literature transcends mere reading ability [31]. Additionally, the health care system is yet to fully leverage social media in disseminating accurate information [32], potentially leaving the majority of the population who depend on this source of information vulnerable to misinformation.

Although this study provides valuable information on COVID-19 vaccine acceptance following the pandemic, it is not without limitations. The survey provided a snapshot of opinions on COVID-19 vaccination in selected districts after the end of the public health emergency, and may not be applicable to the entire region. Therefore, future research should consider longitudinal studies to ascertain how willingness to vaccinate translates into actual vaccination. This would establish the underlying causes of vaccine hesitancy and facilitate institution of remedial actions. Finally, given that several types of vaccines have been administered in Ghana, future studies should consider examining vaccine acceptance based on the types of vaccines.

## Conclusion

This study assessed predictors of COVID-19 vaccination uptake following the declaration of the COVID-19 pandemic as no longer constituting a PHEIC. Although willingness to accept vaccination was high, a considerable proportion of persons who would advocate for COVID-19 vaccination were not willing to take the vaccine themselves. Persons living in urban communities and those with secondary-level education were less likely to accept vaccination, with vaccine hesitancy driven mainly by the fear of anticipated side effects.

While integration of COVID-19 vaccination into routine care would improve uptake, the Savannah Regional Health Directorate should explore tailored communication approaches in urban communities and among persons with secondary-level education, ensuring that highest-risk individuals (health care workers, persons with underlying medical conditions, older adults, and pregnant women) are prioritized. Health education should focus on explaining the pandemic reclassification, the prevailing risk of COVID-19 surge and the implications for health and socioeconomic activities, the importance of continuing vaccination, the typical nature of some side effects (fever, headache, and other constitutional manifestations) of COVID-19 vaccination, and the actions to be taken in the event of adverse effects. Additionally, due diligence must be applied in the engagement of individuals to mobilize communities for vaccination. Prospective advocates must clarify their positions on COVID-19 vaccination, ensuring that they are adequately informed and believe in the messages, and have completed at least the primary series vaccination to prove their commitment to the course. However, this must be managed cautiously to mitigate opposition from persons who might not meet the criteria.

## Declarations of competing interest

The authors have no competing interests to declare.
